# Inequitable walking conditions among older people: examining the interrelationship of neighbourhood socio-economic status and urban form using a comparative case study

**DOI:** 10.1186/1471-2458-10-677

**Published:** 2010-11-05

**Authors:** Theresa L Grant, Nancy Edwards, Heidi Sveistrup, Caroline Andrew, Mary Egan

**Affiliations:** 1 Institute of Population Health, University of Ottawa, 1 Stewart St, Room 300, Ottawa, Ontario, Canada, K1N 6N5; 2 School of Nursing and Department of Epidemiology and Community Medicine, University of Ottawa, 451 Smyth Road, Ottawa, Ontario, Canada, K1H 8M5; 3 School of Rehabilitation Sciences, University of Ottawa, 451 Smyth Road, Ottawa, Ontario, Canada, K1H 8M5; 4 University of Ottawa, School of Political Studies, University of Ottawa, 55 Laurier Avenue East, Room 9101, Ottawa, Ontario, Canada, K1N 6N5; 5 School of Rehabilitation Sciences, University of Ottawa, 451 Smyth Road, Ottawa, Ontario, Canada, K1H 8M5

## Abstract

**Background:**

Supportive neighbourhood walking conditions are particularly important for older people as they age and who, as a group, prefer walking as a form of physical activity. Urban form and socio-economic status (SES) can influence neighbourhood walking behaviour. The objectives of this study were: a) to examine how urban form and neighbourhood SES inter-relate to affect the experiences of older people who walk in their neighbourhoods; b) to examine differences among neighbourhood stakeholder key informant perspectives on socio-political processes that shape the walkability of neighbourhood environments.

**Methods:**

An embedded comparative case study examined differences among four Ottawa neighbourhoods that were purposefully selected to provide contrasts on urban form (inner-urban versus suburban) and SES (higher versus lower). Qualitative data collected from 75 older walkers and 19 neighbourhood key informants, as well as quantitative indicators were compared on the two axes of urban form and SES among the four neighbourhoods.

**Results and discussion:**

Examining the inter-relationship of neighbourhood SES and urban form characteristics on older people's walking experiences indicated that urban form differences were accentuated positively in higher SES neighbourhoods and negatively in lower SES neighbourhoods. Older people in lower SES neighbourhoods were more affected by traffic hazards and more reliant on public transit compared to their higher SES counterparts. In higher SES neighbourhoods the disadvantages of traffic in the inner-urban neighbourhood and lack of commercial destinations in the suburban neighbourhood were partially offset by other factors including neighbourhood aesthetics. Key informant descriptions of the socio-political process highlighted how lower SES neighbourhoods may face greater challenges in creating walkable places. These differences pertained to the size of neighbourhood associations, relationships with political representatives, accessing information and salient neighbourhood association issues. Findings provide evidence of inequitable walking environments.

**Conclusion:**

Future research on walking must consider urban form-SES inter-relationships and further examine the equitable distribution of walking conditions as well as the socio-political processes driving these conditions. There is a need for municipal governments to monitor differences in walking conditions among higher and lower SES neighbourhoods, to be receptive to the needs of lower SES neighbourhood and to ensure that policy decisions are taken to address inequitable walking conditions.

## Background

The importance of walking has received growing attention in recent years in both the fields of public health [1,2] and urban planning [3,4]. Research examining approaches to increase levels of physical activity has shifted from a lifestyle focus to supportive environments for active living [5]. Supportive walking environments are particularly important for older people, as walking is a preferred form of physical activity for a majority of this age group [6-8]. Furthermore, some older adults will face the loss of their driving license because of age-related changes, necessitating other options to meet their transportation needs [9]. Government policy directives to support independent living for older people [10] have created further imperatives for more walkable environments.

The neighbourhood is an important context for older people's walking patterns since this group tends to spend more time in local environments [11-13]. However, walking patterns vary among neighbourhoods [14-16]. Urban form and neighbourhood socioeconomic status (SES) are two dimensions of the neighbourhood environment that have been shown to influence walking behaviour over and above the effects of individual level determinants [17-20]. Urban form refers to the physical layout and design of neighbourhoods (e.g. dwelling density, land use and street patterns), while neighbourhood SES refers to the aggregated social and economic characteristics of the residents (e.g. average neighbourhood income, average level of education, percent of population living in poverty). As a group, residents of lower SES neighbourhoods are more reliant on walking for transportation [21-23], making supportive walking conditions especially important in lower SES neighbourhoods. However, most of the literature on walkability (i.e. the extent to which environments invite and support walking) has tended to focus on either urban form or neighbourhood SES, with less consideration given to how they may inter-relate.

In early studies examining the association between urban form and walking, the high density inner-urban neighbourhoods studied were often low SES areas while the low density suburban areas studied were often high SES areas, making it impossible to disentangle the effects of neighbourhood SES from those of urban form [24]. More recently, this issue has been addressed in a variety of ways. The first has been to select neighbourhoods that vary in urban form while keeping the social characteristics of neighbourhood residents comparable [24-26]. These studies indicated that people walk more in high density inner-urban neighbourhoods than in low density suburban neighbourhoods. However, this approach did not disentangle the effect of neighbourhood environment, often referred to as context, from the compositional effects of individual residents who live in the neighbourhoods. In other words, people may walk more frequently in inner-urban neighbourhoods because a preference for walking influences their choice to live in these types of neighbourhood environments [27,28], or because they are reliant on walking for socio-economic reasons [21-23].

Another approach designed to disentangle context from composition has been to adjust for individual level SES using multilevel models [15,18,19,29-31]. Although the use of these models has allowed simultaneous examination of individual and group level factors, limitations remain [32,33]. One critique of using multilevel models is that individual-level SES '*adjustment' *shifts attention away from what aggregated levels of SES represent at the neighbourhood level. As stated by Diez-Roux [32]:*"Variables measured at the individual-level (such as individual social class or race/ethnicity) may only be meaningfully understood in the context of how individuals are related to each other in groups or societies" *(p.184). Aggregated neighbourhood levels of SES may, therefore, serve as a marker for how groups of people are related in a social hierarchy. These aggregated markers of group SES are often reflected in the characteristics of place, which can constrain health opportunities for lower SES groups [34].

Recent studies using statistical regression models to examine the interaction effects of urban form and SES on walking have provided two particularly relevant insights [28,35]. One is that individual attributes moderate the relationship between the neighbourhood environment and walking behaviour. For example Owen et al. [28] found that individuals who self selected to walkable neighbourhoods walked more weekly minutes compared to those with a lower preference for walkable neighbourhoods. This finding provides support for an ecological view of walking behavior. In other words, there is evidence of an inter-relationship between individual and environmental characteristics. The second important insight is that area-level SES is associated with differences in perceived environmental attributes and partially explains differences in walking across income groups [35].

There is growing evidence of a fundamental inequity among neighbourhoods vis-à-vis the environmental attributes that influence the experience of walking. Lower quality recreational facilities, less pedestrian infrastructure and less green space have been documented in lower SES neighbourhoods compared to high SES neighbourhoods. Similarly, higher levels of perceived traffic noise and crime, and lower levels of perceived aesthetics have been observed in low SES neighbourhoods compared to high SES neighbourhoods [25,26,40,41]. In inner-urban neighbourhoods, the benefits of having destinations in close proximity may be offset by factors such as high vehicular traffic density that make those destinations more hazardous to reach [42,43]. These differences suggest there may be socio-political processes at work that disadvantage lower SES neighbourhoods. Proponents of social justice and health equity [44] argue that addressing systematic patterns of disadvantage is a moral imperative for public health. The distribution of resources and opportunities linked to socio-political engagement ultimately affects health disparities among social groups. Put another way, these resources and opportunities represent collective capacity for creating healthy environments and, therefore, have relevance to public health interventions.

To date, studies examining neighbourhood effects on walking have used a variety of methods and theoretical approaches. Many studies used data from large census tracts as a proxy for neighbourhood-level effects, which did not correspond to meaningful walking distances [45]. A number of authors have developed conceptual frameworks for examining how environmental features may affect walking [46-49]. These frameworks have drawn links between the walking experience and dimensions of the environment, while also proposing ways to operationalize these dimensions. For example, Alphonzo [46] theorized that environmental accessibility influences the decision to walk, and operationalized accessibility in terms of the sidewalk network, barriers to walking, distance to destinations and number of destinations. While the conceptual frameworks developed up to this point have provided useful categorizations for studying the neighbourhood environment and its relation to walking, they fall short in two main ways. The first is in providing guidance for understanding the joint effect of physical and social environments on the walking experience. The second is in providing an understanding of the mechanisms through which disparities in walking conditions among neighbourhoods or communities arise [45,48]. The current study aims to address both shortcomings using a comparative case study approach.

A case study design permits an examination of inter-related dimensions of context such as neighbourhood SES and urban form, and yields findings that support conceptual development [50,51]. Only a few studies on walkability have used this approach. These have examined how citizens' groups acted to affect their local environments [52-54] but focused on externally funded initiatives rather than on neighbourhood processes that occur spontaneously. Furthermore, previous case studies have not examined how the intersection of urban form and neighbourhood SES influence walkability. The current study aimed to examine how urban form and neighbourhood SES may inter-relate to affect the experiences of older people who walk in their neighbourhoods. The study also placed these walking experiences within the socio-political processes that shaped neighbourhood environments and examined differences in how neighbourhood stakeholders described these processes. The design offers a contextualizing process, which is important for characterizing underlying relationships and processes and adding new insights [55].

## Methods

This comparative embedded case study was conducted in four Ottawa neighbourhoods that differed on urban form (inner-urban versus suburban) and SES (higher versus lower). Ottawa, located in the province of Ontario, is the capital of Canada. The main employers are the federal government and the technology sector, resulting in a relatively well educated population. The case was bound by the socio-political structure of Ottawa's municipal organization and the geographic boundaries of neighbourhood embedded units. The study involved sequential collection of qualitative data and quantitative indicators. Phase one examined older people's perspectives on their walking experiences in the four neighbourhoods [56]. Phase two examined the perspectives of neighbourhood key informants who had been involved with neighbourhood actions relevant to walkability issues. Data on publicly available quantitative indicators of amenities relevant to walkability and neighbourhood traffic burden were collected during phase three.

### Sampling and recruitment

#### Neighbourhood selection

Neighbourhoods were purposively selected to vary on urban form and SES. At the time of neighbourhood selection, Ottawa was classified into 50 neighbourhoods. Using sociodemographic data from the 2001 Canadian census, high and low SES neighbourhoods were differentiated on the basis of mean household income, percentage of post-secondary graduates and percentage of low income households. City classifications of urban form were used to divide inner-urban neighbourhoods (primarily established before 1950) from suburban neighbourhoods (primarily established after 1950). Since there were fewer inner-urban than suburban neighbourhoods, the inner- urban neighbourhoods were chosen first to provide the greatest contrast in SES while maintaining comparable percentages of people aged 65 years and above. Subsequently, suburban neighbourhoods were selected to provide comparable SES profiles with inner-urban neighbourhoods. It was verified that all selected neighbourhoods had an active neighbourhood association (also referred to a community association). Table 1 displays the socio-demographic and urban form characteristics of selected neighbourhoods.

**Table 1:** Socio-demographic and urban form characteristics of selected neighbourhoods

Neighbourhood characteristics	Lower SES inner-urban neighbourhood	Higher SES inner-urban neighbourhood	Lower SES suburban neighbourhood	Higher SES suburban neighbourhood
*Older residents (%)	11	9	11	10

*Total population	11947	10630	10106	5237

*Post secondary graduates (%)	51	79	49	73

*Average household income (Canadian $)	41,007	99,313	44,453	108,602

*Low income cut-off households (%)	39	10	35	7

**Dwelling density per square kilometre	3258	1992	1823	840

***Street pattern	Rectilinear grid	Rectilinear grid	Curvilinear; one area with modified grid	Curvilinear street; some cul-de-sacs

Multiple Sources: *City of Ottawa, based on 2001 Canadian Census data

** Kristjansson, E., Sawata, M., & Labonte, R. The Ottawa Neighbourhood Study, based on 2006 Canadian Census data [57]

***City of Ottawa Planning Department

#### Phase one recruitment (older people)

Older people in each of the four study neighbourhoods were recruited through community newspaper advertisements, posters, seniors' centres, community health centres, recreation centres, housing co-ops and apartment buildings. A newspaper with a city-wide distribution included a story about the study, which also facilitated recruitment. Older people were considered eligible to participate if they: 1) lived within one of the neighbourhoods being studied; 2) had lived there for at least 2 years; 3) were 65 years or older and; 4) had walked in their neighbourhood at least once within the past year.

#### Phase two recruitment (neighbourhood stakeholder key informants)

Neighbourhood stakeholders were defined as community members whose actions or decisions have had or potentially could have had an impact on neighbourhood walking conditions. Three types of key informants were purposively recruited. They were people who had lived in, worked in and politically represented the neighbourhood within the previous ten years. Neighbourhood key informants were recruited through neighbourhood community associations, community health centres and contacts made in phase one. Key informants were also asked whether they could recommend other people with relevant knowledge or experience that might be different from their own. Municipal politicians representing the four study neighbourhoods were contacted via e-mail and invited to participate.

### Data collection

#### Phase one qualitative (older people on walking experiences)

Data were collected through focus groups, individual interviews and observational field notes from May 2007 to December 2008. Interviews were semi-structured and designed to elicit discussion on: 1) where people walked and why; 2) supportive and unsupportive aspects of the neighbourhood environment; and 3) positive and negative neighbourhood changes over the past decade that had affected walking. Interviews lasted for approximately 50 minutes and were audio-taped.

#### Phase two qualitative (neighbourhood key informants on community processes)

In-depth interviews were conducted from November 2007 to May 2008. Interviews lasted for approximately 60 minutes and were audio-taped. Participants were asked to describe their insights on the issue of walkability and associated community processes. They were prompted to talk about: 1) the types of individual and group actors; 2) types of issues and how they were addressed; 3) how groups were organized and how they communicated; 4) neighbourhood resources; 5) municipal-neighbourhood interactions; 6) opportunities acted upon.

As data collection progressed during both phases, the researcher (TG) took field notes, which helped to guide subsequent questioning and probes. All data were collected by the lead investigator, a practising physiotherapist working in the field of geriatric and stroke rehabilitation, as part of a doctoral research program in population health. Regular debriefing sessions were held with members of the research team who had backgrounds in epidemiology, nursing, occupational therapy, political science and rehabilitation.

#### Quantitative indicators

Publicly available quantitative indicators relevant to differences in older people's walking experience were identified. Indicators of commercial walking destinations (e.g. presence of grocery stores) and neighbourhood amenities (e.g. walking/cycling paths, parks, recreation centres) were obtained from a website that provided data on neighbourhood health indicators collected by researchers from the University of Ottawa who worked in collaboration with the City of Ottawa [57]. Indicators of neighbourhood traffic burden were collected from the City of Ottawa Public Works and Services Department. These included pedestrian-vehicle collisions, traffic volumes in major intersections and distance of designated trucking routes in the neighbourhood. Total numbers of pedestrian vehicle collisions were summarized for the period January 1, 1998 to January 1, 2007, including collisions that occurred while pedestrians were crossing neighbourhood borders. Major intersections within neighbourhood borders were selected to represent the convergence of a north-south oriented major roadway with an east-west oriented major roadway, and to include intersections described in older people's accounts of traffic hazards in phase one interviews. The intersection data were comprised of traffic and pedestrian counts collected by city staff between the hours of 7:00 a.m. and 6:00 pm over a single weekday. The total length of designated trucking routes per square kilometre was calculated for each of the neighbourhoods using maps available through the City of Ottawa [58] and geographic information system data on neighbourhood areas collected as part of the Ottawa Neighbourhood Study [57].

### Analysis

Phase one and phase two qualitative data were analyzed separately using an inductive and iterative approach. Focus groups and individual interview recordings were transcribed verbatim. The transcripts were read and re-read while the researcher made preliminary notes in the form of memos in the margins of the transcripts. This process facilitated thinking about the data in a holistic contextual manner [59]. Field notes and reflective memos made during data collection were also reviewed. For each transcript, content-oriented summary forms were created that contained information relevant to the questions posed as well as to other emergent topics. A constant comparative analysis method [60] was used. This involved labelling categories within discrete sections of data and comparing data across these categories to identify links, connections and differences. Analysis moved from coding strategies (i.e. categorizing the data) to contextualizing strategies (i.e. considering relationships that linked statements and events within a coherent whole). Re-reading of the transcripts by the lead investigator and subsequent discussion among members of the research team assisted in the integration of categories and conceptual interpretation. Reliability was enhanced by having other members of the research team (ME, CA) verify sections of the transcripts to ensure a credible match between data and coding domains. Authenticity of interpretation was enhanced through feedback received from study participants who were sent a copy of the study results.

**Figure 1:**
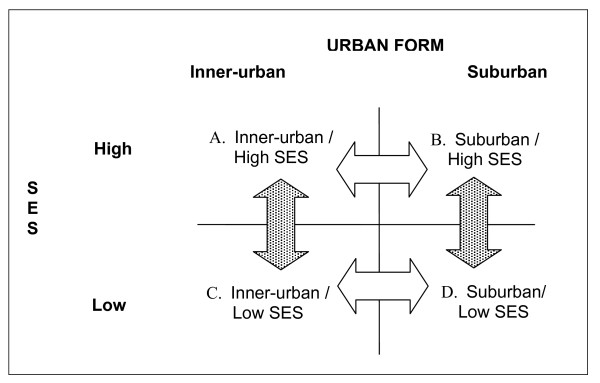
**Comparison strategies**: Horizontal arrows represent urban form comparisons with attention to how differences may be expressed in low and high SES neighbourhoods. Vertical arrows represent SES comparisons with attention to how differences were expressed in inner-urban and suburban neighbourhoods. These comparisons were made within each of the three data sets.

Data from the four neighbourhoods were compared using matrices [61]. Content summary forms were used to construct question- and category-oriented matrices. Conceptually-oriented matrices were constructed based on the findings of phase one and phase two. Differences were further verified using matrices that were specific to subcategories of the broader conceptual dimensions. Figure 1 illustrates the four sets of comparisons that were used to identify differences among the neighbourhoods. These comparisons were made within each of the three data sets using matrices and tables to allow visual display. Figure 1 displays two sets of comparisons made along the urban form axis and two sets of comparisons made along the neighbourhood SES axis. These comparisons permitted an examination of the joint effects of urban form and SES. Looking at how urban form is experienced differently in high and low SES neighbourhoods allowed an exploration of how neighbourhood SES may modify the experience of urban form. Likewise, looking at how the same level of neighbourhood SES may be experienced differently in suburban and inner-urban neighbourhoods allowed an examination of how urban form may modify the experience of neighbourhood SES. Differences identified through qualitative analysis were triangulated with publicly available quantitative indicators.

## Results

Results are presented in three sections. The first provides a summary of participant and neighbourhood characteristics. Section two focuses on urban form while section three concentrates on SES comparisons between inner-urban and suburban neighbourhoods. Finally, a summary table of key qualitative findings and main conclusions after integration with quantitative indicators is presented.

### Section 1: Participant and neighbourhood descriptors

#### Older participants

Three focus groups and three to five individual interviews were conducted in each neighbourhood, yielding 53 focus group participants and 20 individual interviews. The sample included older people who ranged from sedentary (10.2% reported walking rarely and 21.1% reported walking less than 20 minutes per day) to very active (24.9% reported walking five to seven days per week and 7.6% reported walking more than 60 minutes per day). The majority of participants reported walking from one to four days per week (64.9%) and between 20 and 60 minutes per day (71.3%). Table 2 displays the number of and characteristics of older participants in each neighbourhood.

**Table 2:** Sample characteristics of older walkers for each neighbourhood

Sample characteristics	Lower SES inner-urban neighbourhood	Higher SES inner-urban neighbourhood	Lower SES suburban neighbourhood	Higher SES suburban neighbourhood
**Number of participants (n)**	20	17	18	20

**Age (mean years)**	77	77	72	75

**Gender (% female)**	85	76	78	90

**Walking aid use (%)**	35	25	28	10

**Length of neighbourhood residence (mean years)**	15	37	26	28

**Home owners (%)**	10	88	33	75

**Post-secondary education (%)**	25	88	55	45

#### Neighbourhood key informants

Four to six key informants were recruited from each neighbourhood (n = 19; 63% male; 37% female). These participants included present and past members of place-based voluntary groups (n = 12), professionals of place-based institutions (n = 4), and municipal politicians (n = 3). Four older people (one from each neighbourhood) who participated in phase one volunteered to be re-interviewed as key informants because of relevant knowledge.

#### Neighbourhood amenities relevant to walkability

Table 3 illustrates the presence of some neighbourhood amenities relevant to walkability. More banks, pharmacies and grocery stores were present in the inner-urban neighbourhoods than in suburban neighbourhoods. However, only the higher SES inner-urban neighbourhood had any grocery stores. Walking and biking paths as well as park land measures were higher in higher SES neighbourhoods compared to their lower SES counterparts. It is noteworthy that the higher SES inner-urban neighbourhood had higher levels of both walking paths and park land compared to the lower SES suburban neighbourhood. Inner-urban neighbourhoods had a greater number of recreational facilities compared to suburban neighbourhoods. The lower SES inner-urban neighbourhood had the highest number of indoor recreation facilities of all the neighbourhoods but the lowest amount of park land per 1000 residents.

**Table 3:** Neighbourhood amenities relevant to walkability

Amenities	Lower SES inner-urban neighbourhood	Higher SES inner-urban neighbourhood	Lower SES suburban neighbourhood	Higher SES suburban neighbourhood
**Banks (per 1000 residents)**	0.33	0.47	0	0.18

**Pharmacies (per 1000 residents)**	0.55	0.26	0	0

**Grocery Stores (per 1000 residents)**	0	0.19	0	0

**Biking and walking paths (metres per person)**	0.44	1.00	0.71	3.38

**Parks (metres per person)**	2.09	29.88	23.57	93.64

**Recreational facilities (per 1000 residents)**	2.67	2.01	1.23	1.23

Source: Kristjansson, E., Sawata, M., & Labonte, R. The Ottawa Neighbourhood Study. Ottawa Neighbourhood [57], based on 2001 Canadian Census data

#### Indicators of neighbourhood traffic burden

Table 4 provides indicators of neighbourhood traffic burden. Pedestrian vehicle collisions were higher in inner-urban neighbourhoods compared to suburban neighbourhoods. It is noteworthy that inner-urban neighbourhoods also had higher pedestrian:vehicle ratios at major intersections suggesting that collisions may be more likely to occur in these types of neighbourhoods because of higher concentrations of both pedestrians and vehicles. Lower SES neighbourhoods had more than double the number of collisions of their higher SES counterparts over a ten year period. Vehicle volumes in a major intersection were highest in the lower SES suburban neighbourhood. Table 4 also illustrates that lower SES neighbourhoods had greater distances of designated trucking routes per square kilometre compared to high SES counterparts.

**Table 4:** Indicators of neighbourhood traffic burden

Indicators	Lower SES inner-urban neighbourhood	Higher SES inner-urban neighbourhood	Lower SES suburban neighbourhood	Higher SES suburban neighbourhood
*Pedestrian-vehicle collisions (total number)	207	113	78	34

**Vehicle Volume (total number)	11 694	10 554	15 852	7 469

**Pedestrian Volume (total number)	2417	3292	242	122

**Vehicles:Pedestrian (ratio)	4.8 : 1.0	3.2 : 1.0	65.5 : 1.0	61.2 : 1.0

**Designated trucking routes (metres per square kilometre)**	2762	541	831	0

Source: City of Ottawa Public Works and Services Department

* Pedestrian-vehicle collisions 1998 - 2007

** Pedestrian and vehicle volumes for selected main intersections:

Absolute counts collected over an 8 hour weekday

Values for lower SES inner-urban collected July 2007, higher SES inner-urban May 2007, lower SES suburban July 2006, and higher SES suburban July 2006

### Section 2: Urban form comparisons

Two major categories emerged in comparing the walking experiences of inner-urban and suburban older adults across urban form axes: pedestrian infrastructure and walking destinations. The importance of each to older people was further associated with neighbourhood SES as described in the following two subsections. No categorical differences emerged from the urban form comparisons of key informant data.

#### Pedestrian infrastructure

Older people living in inner-urban neighbourhoods more often reported having sidewalks in their neighbourhoods than those living in suburban neighbourhoods. Their discussion of sidewalks highlighted tensions between the liveliness and interest of walking in inner-urban neighbourhoods and the hazards of multiple sidewalk uses. The importance of sidewalks in inner-urban neighbourhoods was associated with safety, since they separated vehicle and pedestrian traffic, as well as with their value in providing frequently used public space (i.e. providing spontaneous meeting opportunities and comfort in knowing others were around to help if required). Since many sidewalks had just been replaced in the higher SES inner-urban neighbourhood much of the discussion in this neighbourhood focused on sidewalk design. The new design, while providing a continuous level portion for the pedestrian on the inner side, had increased the grade of driveway inclines on the outer side and many people found this difficult to navigate especially when walking two abreast in winter. In the lower SES suburban neighbourhood, there was less emphasis on sidewalk design and more emphasis on multiple sidewalk hazards such as carts and fallen fruit on the sidewalks as well as the problem of cyclists and skateboarders using the sidewalks. One key informant noted that while the law prohibited bikes on the sidewalks, many parents were teaching their children to ride on the sidewalks because of concerns about traffic volumes on the roadways:

"They are learning very young not to follow the law. I think it is bad training for the kids. Of course, the parents do not want them on road, so what do you do?"

(Female, phase two interview, lower SES inner-urban neighbourhood)

Suburban discourse on sidewalks highlighted a tension between auto-oriented street design and pedestrian concerns. Lack of sidewalks was perceived to be less of a problem in the higher SES suburban neighbourhood because of lower traffic volumes as well as an extensive network of recreational pathways through green areas. Older people in this neighbourhood felt that the combination of these pathways and green areas was an asset, which invited walking in the neighbourhood. However, they noted the disadvantage of having to share the network with cyclists and the subsequent risk of collisions. They also indicated that the network of pathways was not cleared adequately during winter months. Older people's views in the higher SES suburban neighbourhood revealed, however, that retrofitting suburban areas with sidewalks can be highly controversial:

"We have never missed sidewalks. We always walked on the road and we were quite happy. Well, amalgamation came along and suddenly Penfield was missing a piece of sidewalk and we almost had the third world war about that little piece of sidewalk."

(Female, phase one focus group, higher SES inner-urban neighbourhood)

Key informant interviews indicated that the reluctance to have sidewalks in the higher SES suburban neighbourhood was related to concerns about aesthetics such as how well the sidewalk fits with overall design principles of the neighbourhood, and property values. Opposition to sidewalks was also related to residents' concerns about easily accessing their driveways especially in winter when snow banks made roadways narrower. In other words, the addition of sidewalks was expected to reduce the width of roadways and thus, the space available for manoeuvring vehicles.

Sidewalks were felt to be much more important in the lower SES suburban neighbourhood where vehicle volumes were higher and the availability of recreational pathways was lower than in the higher SES suburban neighbourhood. Many people in this neighbourhood felt that a sidewalk would relieve some of the worry of vehicle hazards and legitimize walking as a form of local transportation:

"I think that one side of a street should have a sidewalk. It is a sign of civility...just so we acknowledge that people walk."

(Female, phase one focus group, lower SES suburban neighbourhood)

In the lower SES suburban neighbourhood, very few of the participants reported using recreational pathways on a regular basis because of having to walk too far to access them or because of concerns about cyclists.

#### Walking destinations

Walking for shopping and errands appeared to be most convenient and enjoyable in the higher SES inner-urban neighbourhood because of having a mix of shops and services nearby, frequent regulated roadway crossings and pleasant surroundings. Older people in the other three neighbourhoods reported that not having a local grocery store was a major factor that reduced the walkability of their neighbourhoods. Older people in the lower SES inner-urban neighbourhood who walked to the nearest grocery store crossed at least three major arterial roads to reach it. Despite a lower number of commercial destinations in the lower SES suburban neighbourhood, many walking destinations were associated with practical errands. In this neighbourhood, people reported walking to shopping centres adjacent to a busy major arterial boarder consisting of six lanes of traffic:

"I do not like Carling and Kirkwood either. There is so much traffic there. There is really not a pedestrian-friendly way to pedal or to walk when you are going to those shopping centers."

(Male, phase one focus group, lower SES suburban neighbourhood)

In the higher SES suburban neighbourhood, people lamented the loss of local commercial destinations but reported that walking paths, beautiful scenery and community destinations like the library and seniors' centre gave them plenty of opportunity and incentive to walk within the neighbourhood. This neighbourhood had the highest amount of park land per person and descriptions of walking destinations emphasized the natural features of the environment. Park areas were associated with the identity and community spirit of this area:

"And this area in here somehow has managed to hang on to its parks. There is the big Samuel Green and God help anybody if they try and build on that. There would be a total uprising."

(Female, phase two interview, higher SES suburban neighbourhood)

### Section 3: SES comparisons

Two major categories emerged on older people's walking experiences when compared across SES axes: traffic hazards and public transit. These issues were experienced differently depending on urban form but generally older people living in lower SES neighbourhoods described greater difficulties with traffic hazards and a greater reliance on public transit. Comparison of key informant descriptions of the socio-political process highlighted four main differences when compared across SES axes. These differences indicated that key informants living in lower SES neighbourhoods described greater challenges in creating walkable places. The following subsections elaborate on these SES differences.

#### Traffic Hazards

Pedestrian-vehicle collisions, vehicular traffic volumes through selected intersections, and designated trucking route distances were higher in lower than higher SES neighbourhoods. Older people in both lower SES neighbourhoods reported having to cross hazardous neighbourhood borders such as main traffic roadways more often to reach desired destinations such as federally maintained parkland and shopping destinations. They also reported more walking hazards associated with heavy vehicles than their higher SES counterparts:

"How they manage to stop when those lights turn to amber, some of those trucks. I get the impression that this city is trying to keep the flow of traffic. The concentration is on the traffic rather than on the pedestrian. That is the feeling that I have."

(Female, phase one interview, lower SES inner-urban neighbourhood)

Older people in the higher SES suburban neighbourhood were more likely to drive to shopping destinations, while those in the higher SES inner-urban neighbourhood reported being able to regularly shop within the neighbourhood and that crossing the main traffic artery within the neighbourhood was not a problem because of frequent regulated pedestrian crosswalks.

Particularly problematic to the lower SES suburban neighbourhood, were long distances between regulated pedestrian crossings on one of the main traffic arteries running through the neighbourhood. High traffic volumes coupled with long block lengths meant that crossing a main arterial in this neighbourhood was difficult. Compounded by a lack of benches, this situation proved especially challenging to older people with mobility problems who lived in the lower SES suburban neighbourhood.

#### Public transit

One topic of conversation that clearly dominated more of the discussion of walkability in lower SES neighbourhoods was the role of public transit. More participants in these neighbourhoods relied on buses in order to complete their walking trips and viewed public transit as integral to walkability. While older people in higher SES neighbourhoods also discussed the importance of public transit for walkability, this observation did not emerge as consistently across focus groups and interviews. Concerns in lower SES neighbourhoods highlighted the importance of providing clear and safe pedestrian connections between pedestrian infrastructure and transit vehicles. Many people talked about the difficultly of having to climb over snow banks to reach the sidewalk after exiting the rear of the bus during winter months. These participants also mentioned several older people who had been killed when they slid under the wheels of the bus after exiting the bus in slippery conditions. Difficulty getting seated before the bus started moving and congestion in priority seating areas were also issues that emerged more frequently in lower SES neighbourhoods. There was a stronger sentiment expressed by older adults in the lower SES suburban neighbourhood than in the lower SES inner-urban neighbourhood that bus routes were not always convenient:

"It is not easy finding a bus that gets you there in an uncomplicated fashion. It is not easy even if you find a bus and are willing to get on it...it is not easy carrying all these baskets of nice new plants."

(Female, phase one interview, lower SES suburban neighbourhood)

#### Socio-political processes

Four differences emerged from comparison of key informants' descriptions of the sociopolitical processes associated with walking issues identified by older people: 1) neighbourhood association size; 2) residence of political representatives; 3) accessing information; 4) salient neighbourhood association issues. Each difference is further described in this subsection.

Higher SES neighbourhoods had **larger neighbourhood association memberships **than their lower SES counterparts. This characteristic appeared to be associated with creating more complex and stable neighbourhood associations in which voluntary work was shared among a greater number of people. In the lower SES neighbourhoods, activities around walkability were addressed by groups with smaller membership numbers:

"It is great having this cultural mix, but it is not only a cultural mix but it is an economic mix and socially mixed, and that is why it is a great neighbourhood. It just does not have the same dynamic as where there is a huge ownership contingent. It puts more burden on a few people but it takes, as Margaret Meade said, it only takes a few people to change the world."

(Male, phase two interview, lower SES inner-urban neighbourhood)

Key informants indicated that **political representatives **for higher SES neighbourhoods lived in those neighbourhoods while political representatives for lower SES neighbourhoods lived elsewhere. Although this did not necessarily mean political representatives misunderstood lower SES neighbourhood issues, it did mean that their own day-to-day walking experiences were often outside the neighbourhoods they were representing, and that there was a greater onus on residents of lower SES neighbourhoods to make them aware of walking issues:

"Then there are other things where I really do not know a lot about it, because I do not live in that area or I am not a senior, so I am not experiencing that current challenge right now.... If I do not know that there is a demand, I am not going to know to ask for it."

(Female, municipal councillor, phase two interview, lower SES suburban neighbourhood)

Residents of higher SES neighbourhoods had the benefit of being represented by councillors whose residency in the neighbourhood gave them a greater appreciation of the issues:

"We have had a very good [councillor] because she is also a resident. She has been a resident for probably close to forty years...you need somebody who knows what your problems are."

(Female, phase two interview, higher SES suburban neighbourhood)

Key informants in higher SES neighbourhoods indicated that groups had been able to generate a strong voice through community associations resulting in improvements to walking conditions in their neighbourhoods. Key informants in lower SES neighbourhood also described having achieved improvements to walkability including pedestrian crossings, and traffic calming measures. However, in the lower SES neighbourhoods, there were more reports of difficulty obtaining information such as building standards or municipal procedures from the City of Ottawa:

"It is very hard to find code. I have been told by various city engineers that say, code is two inches for curb cuts, or that there is no regulation as to the width of the curb cut... They won't give you the information. You have to go research it and find it, nobody will give it to you so that you know whether it meets or it does not meet [the code]."

(Female, phase two interview, lower SES suburban neighbourhood)

The difficulty of **accessing information **did not emerge in conversations with key informants from higher SES neighbourhoods. Although, both groups of key informants described a process of ongoing vigilance, key informants in lower SES neighbourhoods described meeting more resistance as they attempted to navigate municipal level processes. This was the case for neighbourhood group representatives as well as for single residents who acted individually. Although, higher SES neighbourhoods also met with resistance, past successes and the political capacity of their community associations were viewed as factors that seemed to have lowered this resistance at city hall. One resident suggested that city hall may be more receptive to inquiries from the representatives of neighbourhoods with a history of successful community mobilization.

"You do not want to upset five thousand potential voters...I am sure the key committee people, work ten to fifteen hours a week, volunteer, on community business, but it keeps the edge going, it keeps drawing things to people's attention. It is a sad comment that the squeaky hinge gets the oil. We squeak."

(Male, phase two interview, higher SES inner-urban neighbourhood)

Higher and lower SES neighbourhoods differed with respect to **salient neighbourhood association issues **discussed by neighbourhood stakeholder key informants. Key informants in higher SES neighbourhoods gave examples of how neighbourhood association efforts had aimed to improve neighbourhood aesthetics more frequently than in lower SES neighbourhoods. For example, the higher SES inner-urban neighbourhood had organized a series of concerts and raised a considerable amount of money in order to bury hydro-electric wires to improve aesthetics. The higher SES suburban neighbourhood had a heritage committee that was addressing the issue of preserving the original lighting posts in the neighbourhood rather than having a new standard imposed by the city. The light posts along with natural features and winding pathways were felt to contribute to its distinct character, which was part of a comprehensive design plan implemented by the neighbourhood's developer. In the lower SES neighbourhoods there was little mention of aesthetics, but more concern around safety issues (i.e. crime prevention and traffic hazards). In the lower SES suburban neighbourhood, crime prevention activities occurred separately in different parts of the neighbourhood. One key informant from a public housing complex in this neighbourhood described his unsuccessful attempt at organizing a neighbourhood watch initiative. Some of the barriers he faced were raising the funds necessary for advertising the initiative, mobilizing support within his housing community and hearing back from the police in a timely fashion. In contrast, one of the older people in the higher SES inner-urban neighbourhood noted that concerns about crime consumed very few community resources:

"Well that is the only incident [of crime] that I know of... I am a member of the Glebe Community Association. I go to all their meetings once a month and I have been active on the board and it has never been raised in the past five years that I have been going to any meetings."

*(Male, phase two interview, higher SES inner-urban neighbourhood)*.

**Table 5:** Summary of key qualitative findings and main conclusions

Qualitative data set	Urban form thematic differences	Neighbourhood SES thematic differences	Conclusions
**Older people's** **walking** **experiences**	Pedestrian infrastructure Walking destinations	Traffic hazards Public transit	Advantages and disadvantages of urban form were accentuated positively in higher SES neighbourhood and negatively in lower SES neighbourhoods.

**Key informant** **descriptions of** **the socio-political** **process**		Size of neighbourhood association groups Residence of political representatives Accessing information Salient issues: safety versus aesthetics	Lower SES neighbourhood key informants described greater challenges in creating walkable neighbourhoods than their higher SES counterparts.

## Discussion

### The inter-relationship of neighbourhood SES and urban form: compounding effects on older people's walking experiences

Examining the inter-relationship of neighbourhood SES and urban form characteristics with older people's walking experiences highlighted the role of compounding effects - some positive and some negative. On the positive side, for example, a combination of extensive walking paths, abundant and well-kept park space and key community destinations made walking an inviting and pleasant experience in the higher SES suburban neighbourhood despite the lack of commercial destinations. Similarly, a combination of commercial destinations, pleasant surroundings and neighbourhood traffic calming approaches made walking especially convenient in the higher SES inner-urban neighbourhood. Conversely, in the lower SES suburban neighbourhood, the combination of a lack of pedestrian infrastructure, greater distances between walking destinations and a greater reliance on walking and public transit for transportation made walking inconvenient for many participants who lived in this neighbourhood. Although pedestrian-traffic collisions occurred in all four neighbourhoods, the number of collisions was especially high in the lower SES inner-urban neighbourhood. This finding is consistent with those of LaScala, Gerber & Gruenewald [43] who identified *"hot spots" *of pedestrian-vehicle collisions in socially disadvantaged inner-urban neighbourhoods. The compounding effects of having more residents who rely on walking for transport in lower SES neighbourhoods and higher volumes of traffic may partially explain this finding.

In essence, the compounding effects highlighted by this study suggest that there are advantages and disadvantages associated with urban form but that these differences are accentuated positively in higher SES neighbourhoods and negatively in lower SES neighbourhoods. Comparisons of neighbourhood walking amenities provided support for this conclusion. With the exception of pharmacies and recreational facilities, the levels of all other amenities relevant to older people's walking experiences were higher in higher SES neighbourhoods compared to their lower SES counterparts. Although older participants in both inner-urban neighbourhoods appreciated having a mix of commercial walking destinations, it was participants of the lower SES inner-urban neighbourhood who expressed that the absence of a grocery store represented a disadvantage in terms of how they thought about walkability. Comparisons of park land as well as walking and biking paths also demonstrated clear differences between higher and lower SES neighbourhoods. Although some research has suggested that higher levels of green space and recreational pathways might be found in suburban neighbourhoods [62,63], it is notable that both types of amenities were more accessible in the higher SES inner-urban neighbourhood compared to the lower SES suburban neighbourhood in the current study. This finding indicates that neighbourhood SES may be a stronger determinant of certain walking amenity levels than urban form.

Differences found in both older people's descriptions of their walking experiences and the quantitative indicators of traffic burden provide additional support for the compounding effects produced by the inter-relationship of urban form and neighbourhood SES. Concerns with traffic hazards were described by all older participants but to a much greater extent in the lower SES neighbourhoods. These differences were also reflected in quantitative indicators of traffic burden. The suburban differences between the number pedestrian-vehicle collisions and intersection vehicle volumes may, in part, be explained by a higher population in the lower SES suburban neighbourhood. However, there was a clear difference in both these indicators demonstrated in the inner-urban neighbourhoods, which have comparable populations. Furthermore, the differences in distances of designated trucking route demonstrated contrasts in higher and lower SES neighbourhoods that would not necessarily be relevant to population differences.

The question that arises from these findings is whether the neighbourhood SES differences highlighted by this research reflected material differences driven by market forces, or whether they reflected systemic differences in socio-political processes. It could be argued that greater traffic hazards in lower SES neighbourhoods may partly be explained by real estate market forces since, for example, it is generally less costly to live next to a busy roadway than to a park. However, the problems associated with living next to a heavily trafficked area are created and sustained by transportation policy decisions. Furthermore, differences in key informants' descriptions of the socio-political processes emerged in SES neighbourhood comparisons rather than in urban form comparisons. While the latter finding does not necessarily mean that SES differences in walking conditions were created by the particular socio-political processes identified, it does indicate that socio-political processes can have a role in perpetuating these differences. The following section considers the socio-political process differences identified in this study and provides an interpretation of how they may be contributing to inequitable walking conditions.

### Socio-political differences

Results suggest that lower SES neighbourhoods may face greater challenges in creating walkable places. The first of these challenges relates to the role and the critical mass of neighbourhood associations. Although, neighbourhood associations operate in conjunction with a variety of other stakeholders (e.g. community based agencies, tenant organizations, local businesses, schools and local associations), they are often the most recognizable group associated with the neighbourhood as a whole [64,65]. Previous research indicates that home owners are more likely to participate in neighbourhood improvement organizations than individuals who rent [66,67] and that higher SES neighbourhoods have greater levels of home ownership [68,69]. Thus, neighbourhoods with lower numbers of homeowners are often associated with smaller neighbourhood associations. Furthermore, because membership is usually associated with a monetary fee, larger and wealthier community associations can generate more financial resources, which can be used to enhance organizational capacity, thereby increasing membership and allowing options such as hiring external consultants. There is evidence that larger groups are more effective politically [70,71].

The second challenge related to relationships with political representatives. The role of local political representatives may be particularly important for creating walkable neighbourhoods in decentralized municipal systems, since there is no centralized executive body to override political decisions taken with respect to one constituency. Findings from the current study suggest that when a local political representative lives in the neighbourhood, they possess additional knowledge on that neighbourhood through their own walking experiences and daily observations. When they live elsewhere, residents must take the extra step of ensuring that the political representative remains up-to- date on neighbourhood issues. Future research may test the implications of this observation by examining whether improvements are more likely to occur in neighbourhoods with elected politicians residing in them. A rival hypothesis regarding the importance of where councillors live, is the importance of the relationship that neighbourhoods forge with their political representatives. Radoki [72] found that despite a commitment to poverty reduction, urban politics are rarely responsive to the needs of the poor. Based on her case study work in 10 developing cities, she concluded that gains by low income urban residents were achieved by fostering relationships with elected representatives at various levels.

The third set of challenges for lower SES neighbourhoods identified by key informants related to greater difficulties obtaining information about municipal procedures and standards. Whether this difference was relevant to the knowledge base of key informants, the size of the group making the request or the perceived legitimacy of the concern could not be determined from the interviews. Although previous research has indicated that municipal administrators' receptivity to citizens' contacts may be low in general [73,74], there is evidence to suggest that the perceived legitimacy of a neighbourhood issue in the eyes of municipal officials is affected by the level of mobilization and resident participation that occurs around that issue, which often is often higher in affluent neighbourhoods [64,75-77]. The extent to which municipal receptivity and perceived legitimacy may vary with respect to socially advantaged and disadvantaged neighbourhoods has important implications for creating walkable environments and warrants further study.

The final socio-political difference found in this study, relating to the salience of neighbourhood association issues, highlights a further challenge that lower SES neighbourhood may face in creating walkable environments. That is -a greater gap between neighbourhood problems and the resources available to deal with them in lower SES neighbourhoods compared to their higher SES counterparts. For example, higher SES neighbourhood key informants talked about organizing around aesthetic improvement projects, while lower SES neighbourhood key informants talked about organizing around safety. If lower SES neighbourhoods must expend organizational resources on basic concerns that make walking safe (i.e. crime and traffic control) there may be fewer resources left over to address issues that make walking inviting (i.e. aesthetics).

The differences identified in the current study do not establish a clear relationship between neighbourhood SES and the socio-political processes around walkability. However, they highlight the need for further research and analysis regarding this relationship. There have been a number of conceptual approaches used to interpret differences in socio-political processes on health outcomes including differences in a neighbourhood's place within a larger political structure and ideology [78] as well as sources of social capital [79-81]. Although the results of the current study do not fall completely into one conceptual model, findings support the importance of further research that examines both the relationships that neighbourhood groups establish across neighbourhood boundaries (i.e. role of vertical and bridging social capital) as well as examining a larger ideological context that equates property ownership with citizenship [82,83].

### Equitable walking environments

Taken together, the findings discussed in the previous two sections provide insights on the issue of equitability as it relates to walkable neighbourhoods. The concept of health equity, as it is defined from a social justice and human rights perspective, emphasizes the need to look at the distribution of health and living conditions as well as the socio-political processes driving health disparities among socially advantaged and disadvantaged groups [44,84]. Examining how neighbourhood SES and urban form inter-relate to affect older people's walking experiences highlighted clear differences between lower and higher SES neighbourhoods, pointing to a social gradient of walkability. However, looking at these differences devoid of socio-political processes does not go far enough in explaining inequity. Socio-political process differences indicate that the social gradient represents more than material differences. They also indicate that the problem of unequal distribution of walkability among neighbourhoods is unnecessary, and amenable to intervention.

Potential interventions to address inequitable walking conditions could include measures that support neighbourhoods in community mobilization efforts as well as mechanisms to ensure that concerns from lower SES are addressed at the municipal level. Other examples include municipal systems that monitor unequal neighbourhood conditions as well as policy and implementation strategies to reduce inequities through city-wide approaches. The challenges, however, of implementing a city-wide approach to increasing walkability, will inevitably be met by resistance from other interests such as from those who depend on automobile transport.

Since automobile-dependence is widespread, particularly in North American cities [85], the inherent challenge of creating more walkable cities and reducing neighbourhood inequity is apparent. However, a growing recognition of the need for more sustainable transportation systems supports the imperative to address these challenges [86]. Resolving tensions that arise in cities among pedestrian and automobile drivers will require mechanisms that bring diverse interests together and ensure that socially disadvantaged groups have a meaningful voice in this process. Promising examples include approaches being used by the Study Circle Resource Center [87] and the Canadian Institute for Public Engagement [88], which employ a number of strategies including small working groups, the use of personal stories and deliberative dialogue techniques to ensure that a diverse range of citizens can participate and reach decisions on disagreements. As stated by McCoy and Scully [87]:

To become engaged people need to see that their participation will make a difference and that it will be valued. They need opportunities that allow them to make the best use of their skills and time. They need to be invited to participate by those they know and trust (2002, p. 120).

The current study highlights a need for developing further public dialogue around inequitable walking conditions and employing strategies to ensure that there is a place for everyone in this process.

### Implications for the measurement of walkability

The development of walkability measures to date has focused on differentiating between built environment characteristics found in higher density inner-urban neighbourhoods and lower density suburban neighbourhoods. Findings from the current study suggest that additional factors must be taken into consideration for a more accurate picture of how neighbourhood environments support walking. Such factors include vehicular volume, designated trucking route distances and pedestrian/vehicle collisions. These factors could become part of a composite index reflecting traffic burden, which could also contain other relevant measures such as vehicular speeds and air quality. Differences observed in the current study suggest that measures capturing the ratio of pedestrian infrastructure, including recreational pathways, to traffic burden may be useful in differentiating levels of walkability among suburban neighbourhoods. In other words, suburban neighbourhoods may be comparable for proximity of destinations, but walking amenities and hazards may vary greatly.

Furthermore, this study demonstrated that older people experience walking as one part of an integrated transportation system. If walking is to be a practical method of reaching destinations for older adults, public transportation must be easily accessed by walking routes. Therefore, any assessment of walkability must include how well pedestrian infrastructure interfaces with public transit. Finally, in areas with seasonal variation in surface conditions, walkability indices must take these into consideration.

### Considerations for transferability and limitations

This study was conducted in Canada's national capital. Findings must be interpreted within the context of an economically prosperous city with 20% of its total land use devoted to parks and green space [89]. Although the selected neighbourhoods differed with respect to documented rates of personal crime [57], these differences were not reflected in older people's descriptions of their walking experiences in high and low SES neighbourhoods. This finding contrasts with other research [14,25] and may be explained by the relatively low levels of major crime in the city as a whole [90]. It is also possible that the older persons who volunteered for this study may have been less fearful of crime than the population of older residents.

The majority of participants in this study walked moderately and regularly in their neighbourhoods. A smaller segment walked occasionally but all participants had walked at least once in their neighbourhoods in the year prior to being interviewed. This selection approach leaves out the perspectives of non-walkers who may be disabled and/or deterred by neighbourhood conditions. Since environmental conditions can create disability for some individuals [91], future research should consider the perspectives of these non-walkers.

The indicators of traffic burden used did not represent measures of overall traffic volume nor did they represent measures of pedestrian risk. Selected intersection volumes were not all collected during the same year but these were the most recent and comparable data available from the City of Ottawa when this study was conducted. Despite this limitation, these values provided an indication of how major intersection traffic volumes may differ between higher and lower SES neighbourhoods. Although findings were particular to the four neighbourhoods selected for this study, the differences among neighbourhoods provided insights on the broader concept of equity, which is applicable to other places and future research.

## Conclusion

Examining the inter-relationship of neighbourhood SES and urban form with respect to older people's walking experiences indicated walking conditions were more supportive in higher SES neighbourhoods compared to their lower SES counterparts. Socio-political process differences emerged in comparisons of SES rather than urban form comparisons, indicating that differences in walkability among higher and lower SES neighbourhoods are not only unequal but inequitable. Future research on walkability must consider the urban form-SES inter-relationship and further examine the equitable distribution of walking conditions as well as the as the socio-political processes driving these inequities. Study findings highlight the need for municipal governments to monitor differences in walking conditions among advantaged and disadvantaged neighbourhoods, to be receptive to the needs of disadvantaged neighbourhoods and to ensure policy decisions are taken to reduce inequitable walking conditions.

## Declaration of competing interests

The authors declare that they have no competing interests.

## Authors' contributions

TG conceived of the study and designed it in collaboration with ME, NE and HS. TG collected and coded the data. TG, NE, HS, CA and ME were involved with data analysis. TG drafted the manuscript and all authors were involved with subsequent revisions. All authors read and approved the final manuscript.

## Pre-publication history

The pre-publication history for this paper can be accessed here:

http://www.biomedcentral.com/1471-2458/10/677/prepub
